# Supplementary material for: Nano and Microemulsions for the Treatment of Depressive and Anxiety Disorders: An Efficient Approach to Improve Solubility, Brain Bioavailability and Therapeutic Efficacy
Source: Pharmaceutics. 2022 Dec 16;14(12):2825. doi: 10.3390/pharmaceutics14122825 (PMC9783281; doi:10.3390/pharmaceutics14122825)
Supplement: Supplementary file 1 [file pharmaceutics-14-02825-s001.zip › pharmaceutics-2086767-supplementary.pdf]

pharmaceutics

MDPI

Supplementary Materials: Nano and microemulsions for the treatment of depressive and anxiety disorders: an efficient approach to improve solubility, brain bioavailability and therapeutic efficacy

Patrícia C. Pires, Ana Cláudia Paiva-Santos and Francisco Veiga

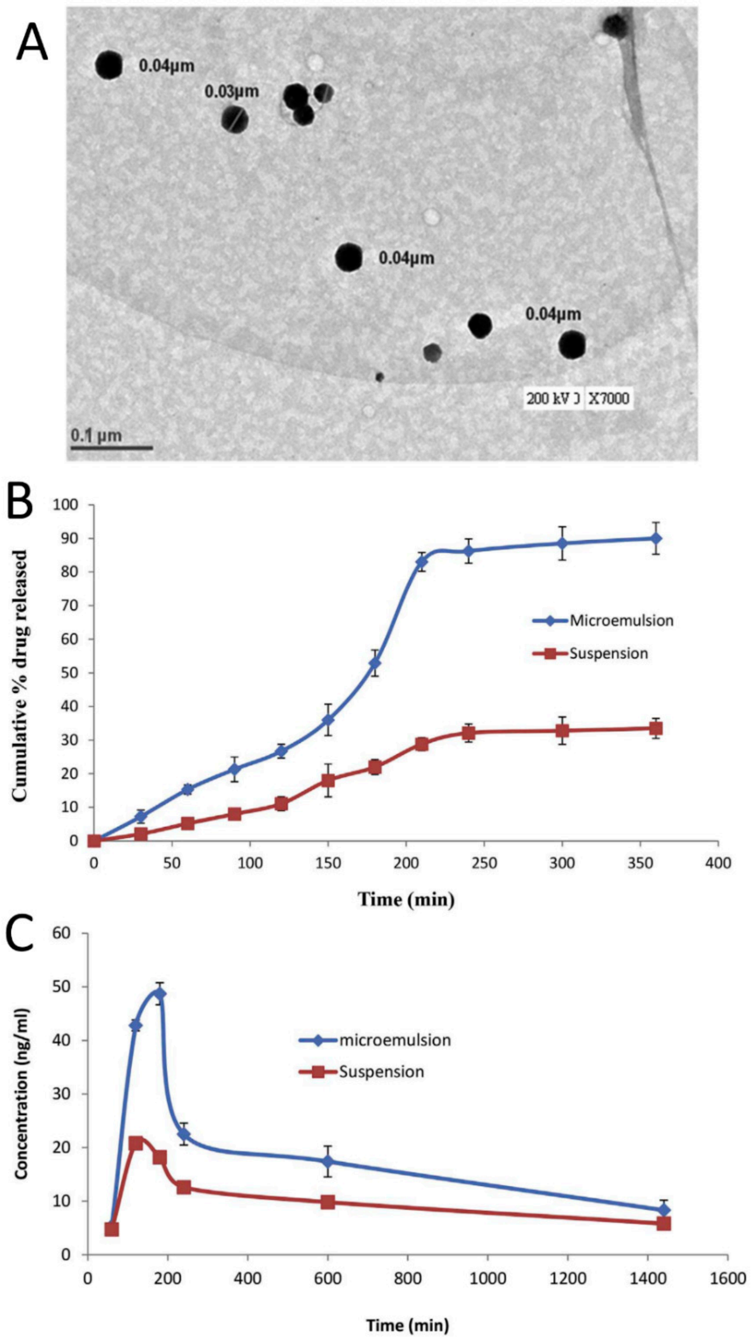

Figure S1. A – Transmission electron microscopy image of the optimized duloxetine microemulsion; B – Cumulative drug permeation profiles of the developed duloxetine microemulsion and a

duloxetine suspension; C - Plasma drug concentration vs time profile of the developed duloxetine microemulsion and duloxetine suspension; adapted from Sindhu *et al.* [41], reproduced with permission from Elsevier [License Number 5430881485045].

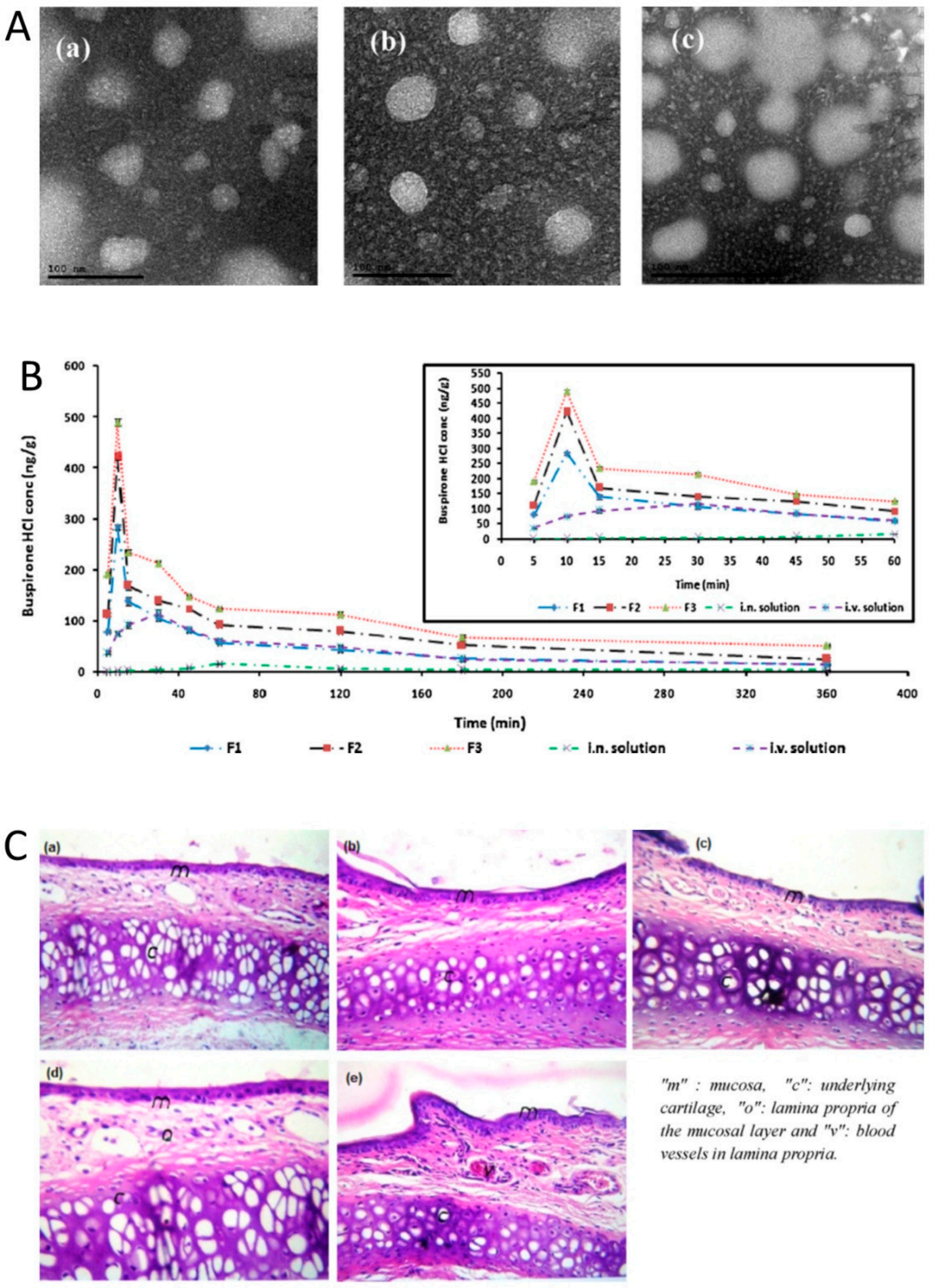

Figure S2. A – Transmission electron microscopy images of the developed buspirone (hydrochloride) microemulsions F1 [non-mucoadhesive, (a)], F2 [mucoadhesive, (b)] and F3 [mucoadhesive with cyclodextrins, (c)]; B - Mean buspirone (hydrochloride) concentrations, in rat brain, after intranasal administration of the developed microemulsions [F1 (non-mucoadhesive), F2 (mucoadhesive) or F3 (mucoadhesive with cyclodextrin)], an intranasal drug solution or an intravenous drug solution; C - Light photomicrograph of an untreated rat nasal epithelium (a), and rat epithelium treated with normal saline pH 6.8 (b), or the developed buspirone (hydrochloride) microemulsions, F1 [non-mucoadhesive, (c)], F2 [mucoadhesive, (d)] and F3 [mucoadhesive with cyclodextrins, (e)]; adapted from Bshara *et al.* [47], reproduced with permission from Elsevier [License Number 5430180908852].

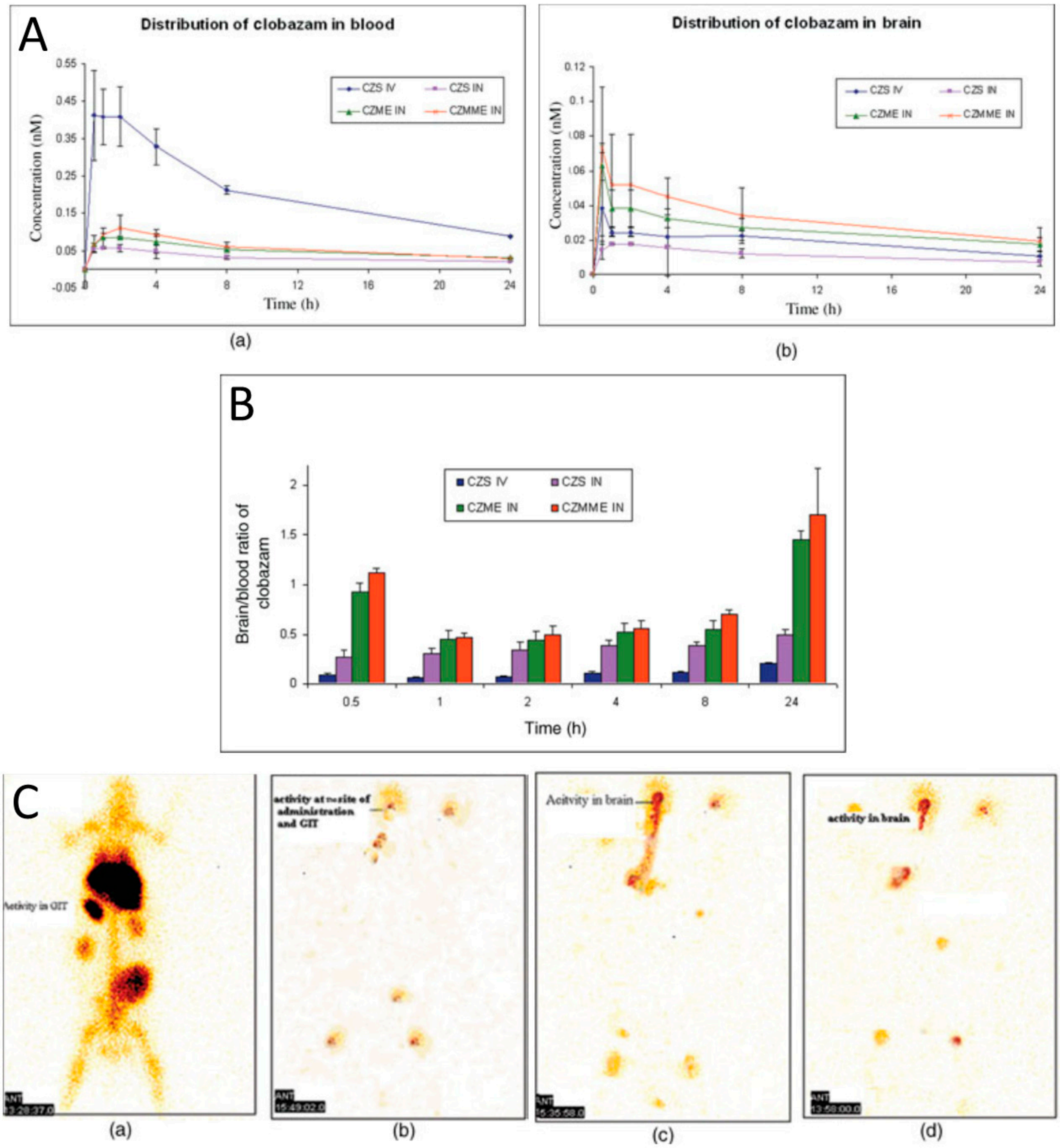

Figure S3. A – Mice blood (a) and brain (b) clobazam concentrations after intranasal administration of the developed non-mucoadhesive microemulsion (CZME IN), mucoadhesive microemulsion (CZMME IN) or drug solution (CZS IN), or intravenous administration of a drug solution (CZS IV); B – Brain/blood ratios of clobazam concentrations after intranasal administration of the developed non-mucoadhesive microemulsion (CZME IN), mucoadhesive microemulsion (CZMME IN) or drug solution (CZS IN), or intravenous administration of a drug solution (CZS IV), at all studied time points; C - Gamma scintigraphy images, in rabbits, after intravenous administration of a drug solution (a), intranasal administration of a drug solution (b), or intranasal administration the developed non-mucoadhesive microemulsion (c) or mucoadhesive microemulsion (d); adapted from Florence *et al.* [49], reproduced with permission from Elsevier [License Number 5430190254854].
